# Benzodiazepine use during an IVF cycle and its impacts on miscarriage and other pregnancy outcomes

**DOI:** 10.1093/hropen/hoag065

**Published:** 2026-07-17

**Authors:** HyunJoo Lim, Eun-Young Choi, Yongtai Cho, Hyesung Lee, Azar Mehrabadi, Jung Yeol Han, Seung-Hwan Lee, Ju-Young Shin

**Affiliations:** Department of Biohealth Regulatory Science, Sungkyunkwan University, Suwon, South Korea; School of Pharmacy, Sungkyunkwan University, Suwon, South Korea; Centre for Pharmacoepidemiology, Division of Clinical Epidemiology, Department of Medicine Solna, Karolinska Institutet, Stockholm, Sweden; School of Pharmacy, Sungkyunkwan University, Suwon, South Korea; Department of Medical Informatics, Kangwon National University College of Medicine, Chuncheon, South Korea; Department of Obstetrics & Gynecology and Pediatrics, Dalhousie University, Halifax, NS, Canada; Korean Mothersafe Counselling Center, Department of Obstetrics and Gynecology, Inje University, Ilsan Paik Hospital, Goyang, South Korea; Clinical Emotion and Cognition Research Laboratory, Inje University, Goyang, South Korea; Department of Psychiatry, Inje University, Ilsan Paik Hospital, Goyang, South Korea; Bwave Inc, Goyang, South Korea; Department of Biohealth Regulatory Science, Sungkyunkwan University, Suwon, South Korea; School of Pharmacy, Sungkyunkwan University, Suwon, South Korea; Department of Clinical Research Design & Evaluation, Samsung Advanced Institute for Health Sciences and Technology (SAIHST), Sungkyunkwan University, Seoul, South Korea

**Keywords:** IVF, benzodiazepine, clinical pregnancy, miscarriage, live birth, preterm birth

## Abstract

**STUDY QUESTION:**

How does benzodiazepine use during an IVF cycle affect pregnancy outcomes?

**SUMMARY ANSWER:**

Benzodiazepine use was neither positively nor adversely associated with IVF pregnancy outcomes overall.

**WHAT IS KNOWN ALREADY:**

Benzodiazepine use during oocyte retrieval, primarily with midazolam, and diazepam use at the time of embryo transfer (ET) have been reported to yield comparable procedural success rates to those of non-users, respectively. However, these studies focus on exposure at a single time point, necessitating a more comprehensive investigation of benzodiazepine use across the entire IVF treatment period.

**STUDY DESIGN, SIZE, DURATION:**

A retrospective cohort study was conducted using Korea’s Health Insurance Review and Assessment (HIRA) database between October 2017 and June 2024. A total of 99 484 women with a first completed IVF cycle were included.

**PARTICIPANTS/MATERIALS, SETTING, METHODS:**

Benzodiazepine users were defined as individuals who had at least one prescription during an IVF cycle (from the last menstrual period to ET) (user group, n = 28 649). Non-users were defined as those who had no benzodiazepine prescriptions within 365 days prior to ET to minimize exposure misclassification (non-user group, n = 61 433). The outcomes of interest were four IVF outcomes: clinical pregnancy rate, miscarriage rate, live birth rate, and preterm birth rate. To compare the two groups, propensity score overlap weighting was applied to adjust for potential imbalances in confounders. Relative risks (RRs) with 95% CIs were estimated using Poisson regression in the overlap-weighted at-risk population cohort for each outcome.

**MAIN RESULTS AND THE ROLE OF CHANCE:**

Compared with non-users, benzodiazepine users had a lower prevalence of prior live birth and a higher prevalence of anxiety disorders, along with greater use of antidepressants before adjustment. The absolute event rates of each outcome were as follows: (i) clinical pregnancy rate: 36.0% of users versus 35.7% of non-users, (ii) miscarriage rate: 14.9% versus 15.2%, (iii) live birth rate: 99.3% versus 99.3%, (iv) preterm birth rate: 13.3% versus 11.6%. Most IVF outcomes were comparable between groups, though a marginally increased risk of preterm birth was observed among users (clinical pregnancy: adjusted RR 1.02 [95% CI 1.00 to 1.05]; miscarriage: 0.99 [0.91 to 1.08]; live birth: 1.00 [1.00 to 1.00]; preterm birth: 1.10 [0.99 to 1.21]). However, stratified analyses by cumulative dose showed no dose–response relationship (≤1.2 mg and >2 mg, respectively—preterm birth: 1.09 [0.99 to 1.21] and 1.01 [0.87 to 1.17]). Additionally, the point estimate was closer to the null among users prescribed both injectable and oral agents during the cycle (preterm birth: 1.04 [0.86 to 1.26]). The robustness of the main findings was supported by analyses accounting for benzodiazepine exposure during pregnancy and by multiple sensitivity analyses.

**LIMITATIONS, REASONS FOR CAUTION:**

Despite rigorous statistical adjustment, residual confounding by unmeasured factors and potential exposure misclassification due to discrepancies between prescription timing and actual medication use cannot be completely ruled out.

**WIDER IMPLICATIONS OF THE FINDINGS:**

This large population-based study found no association between benzodiazepine use throughout the IVF cycle and pregnancy outcomes. Although a small increase in preterm birth risk was observed, there was no evidence of a dose–response relationship or additional risk among patients with exposure to both oral and injectable formulations during the exposure assessment period. However, further studies are needed to evaluate the potential association.

**FUNDING:**

This work was supported by the National Research Foundation of Korea (NRF) grant funded by the Korea government (MSIT) (RS-2026-25474077); This research was supported by a grant (RS-2026-25511535) from Ministry of Food and Drug Safety in 2026.

**DISCLOSURES:**

J-YS received grants from the Ministry of Food and Drug Safety, the Ministry of Health and Welfare, the National Research Foundation of Korea, and pharmaceutical companies, including LG Chem, GSK, Pfizer, Handok, JnJ, and SK Bioscience, outside the submitted work.

**TRIAL REGISTRATION NUMBER:**

N/A.

WHAT DOES THIS MEAN FOR PATIENTS? As infertility becomes more common, the number of women undergoing IVF has increased to achieve pregnancy. An IVF cycle consists of a series of treatments and procedures, including hormone therapy to stimulate ovulation, egg retrieval, fertilization, and embryo transfer. During the process, benzodiazepines can be used to help patients relax before procedures, reduce anxiety, and decrease uterine contractions. However, it remains unclear whether the use of these medications throughout the treatment period has a positive or negative impact on pregnancy outcomes. Therefore, this study investigated the effect of benzodiazepine use during the IVF cycle and found no association with pregnancy outcomes. While a marginal increase in the risk of preterm birth was observed, its magnitude was small. Nevertheless, given the slightly increased risk observed in certain subgroups with specific characteristics, careful consideration of patients’ baseline obstetric and clinical characteristics is warranted when determining whether to use benzodiazepine.

## Introduction

Infertility has emerged as a significant global public health concern, with ∼18% of individuals of reproductive age affected as of 2022 (World Health Organization, 2023). In parallel with this trend, the use of ART, particularly IVF, has expanded rapidly over the past few decades (Horton *et al.*, 2022).

IVF is an ART procedure in which fertilization occurs outside the body, and oocyte retrieval is an essential step in this process. However, oocyte retrieval can induce pain and anxiety; therefore, conscious sedation combined with analgesia is commonly administered in clinical practice (Kwan *et al.*, 2006). In this context, benzodiazepines are often used in combination with propofol and/or opioids, contributing to the reduction of peri-procedural psychological stress through their anxiolytic and hypnotic effects (Trout *et al.*, 1998; Matsota *et al.*, 2015; Ulyatt and Martins da Silva, 2022). In addition, the smooth muscle-relaxant effects of benzodiazepines during embryo transfer (ET) may suppress uterine contractions and thereby facilitate endometrial implantation (Bulletti and de Ziegler, 2005; Zhu *et al.*, 2014; Bellver and Simón, 2018). Beyond their procedural use, benzodiazepines are widely prescribed for the treatment of anxiety disorders, stress-related conditions, and insomnia (Brunton *et al.*, 2018), and may therefore be used to manage patients’ psychological status throughout the IVF cycle. In this regard, the pharmacological properties of benzodiazepines may extend to the entire IVF process, thereby potentially contributing to improved reproductive outcomes (D’Hulst *et al.*, 2009; Kalluru *et al.*, 2021).

However, repeated or cumulative exposure to benzodiazepines may lead to drug accumulation in follicular fluid, potentially affecting oocyte quality (Van Wemmel *et al.*, 2005; Matsota *et al.*, 2015), and may also influence early embryonic cell proliferation and differentiation (Lee *et al.*, 2004). Despite this ambiguity, few observational studies have examined this topic and have primarily evaluated single-dose diazepam exposure at the time of ET, with no significant improvement in pregnancy outcomes observed (Kalluru *et al.*, 2021; Brandão *et al.*, 2024). Therefore, this study aimed to evaluate the association between benzodiazepine use and IVF outcomes, accounting for comprehensive exposure during the IVF treatment period.

## Materials and methods

### Ethics approval

This study protocol was approved by the Institutional Review Board of Sungkyunkwan University, South Korea (No. 2025-06-052), and the requirement for informed consent was waived.

### Study design and data source

We designed this retrospective cohort study by emulating a hypothetical pragmatic randomized trial, an approach that aims to reduce potential biases arising from design flaws (Hernán and Robins, 2016; Hernán *et al.*, 2022). Following the key components of this framework, we developed a protocol to address a causal research question using the Health Insurance Review and Assessment (HIRA) database (Supplementary Table S1). The HIRA database is highly representative of the entire Korean population (≥50 million) and is managed by the government. It contains comprehensive information on demographic and socioeconomic characteristics, as well as in- and outpatient healthcare utilization, including diagnoses, procedures, and prescribed medications, until individuals are disqualified due to emigration or death. This study adhered to the Strengthening the Reporting of Observational Studies in Epidemiology (STROBE) reporting guideline (von Elm *et al.*, 2007).

### Study population and benzodiazepine use

**Figure 1.**
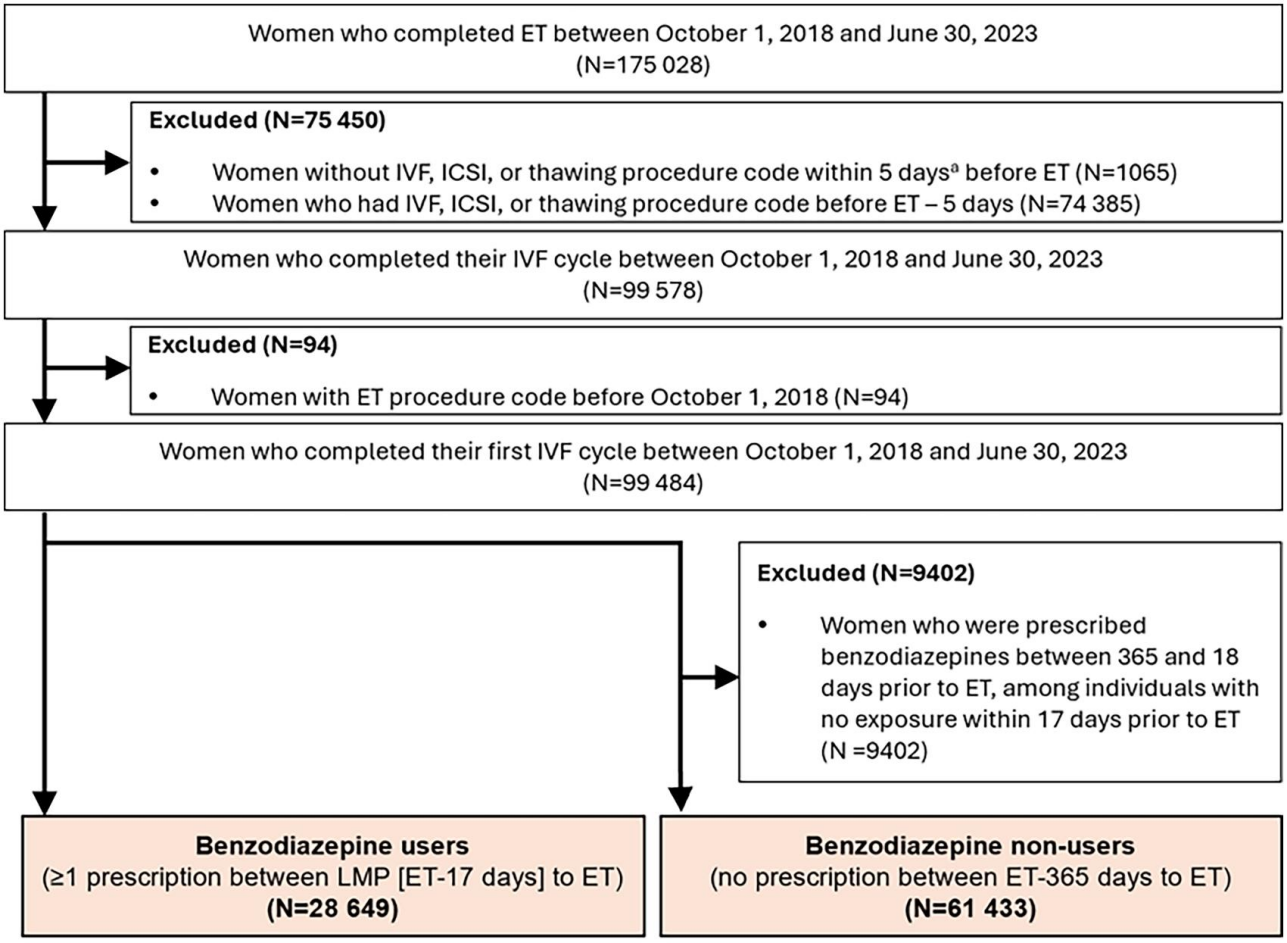
**Flowchart of study cohort.** ET, embryo transfer; LMP, last menstrual period. ^a^To identify the first IVF cycle that progressed to ET, we included only cycles that involved IVF, ICSI, or thawing procedures followed by ET within 5 days, in alignment with the standard IVF cycle schedule.

In South Korea, insurance coverage for infertility treatment procedures was implemented on 1 October 2017, so only cycles performed after this date were identifiable in the database. Accordingly, we defined the index period from 1 October 2018 to 30 June 2023, incorporating a 1-year washout period to increase the probability of inclusion of each woman’s first completed IVF cycle (Supplementary Fig. S1). Cycles that did not reach ET were excluded due to the potential impact of male factor infertility on failed fertilization, which could introduce bias (Cesta *et al.*, 2016). Lastly, we classified them into benzodiazepine user and non-user groups based on whether they had at least one benzodiazepine prescription between the last menstrual period (LMP, defined as ET − 17 days) and ET. However, as exposure was determined based on prescription records, we could not completely rule out the possibility that medications prescribed before the exposure window were used during that period. This may have led to exposure misclassification bias. Therefore, we excluded individuals who had received benzodiazepine between 365 and 18 days before ET from the non-user group (Fig. 1). The benzodiazepines included in the study are listed in Supplementary Table S2.

### IVF outcomes

We examined the rates of four IVF outcomes: clinical pregnancy, miscarriage, live birth, and preterm birth. Clinical pregnancy was defined based on the presence of a first-trimester ultrasound procedure code within 4 weeks after ET. To ensure the validity of the outcome, specific cases were excluded from clinical pregnancies if they met any of the following criteria: (i) absence of a pregnancy episode; (ii) ectopic pregnancy; (iii) a pregnancy end date earlier than the ultrasound procedure date; or (iv) the presence of ART-related procedure records (e.g. IVF, ICSI, IUI, oocyte retrieval, ET, or thawing) between the ultrasound procedure date and the pregnancy end date. Pregnancy episodes were identified using relevant procedure or diagnosis codes within outcome-specific time windows based on a hierarchical algorithm. This algorithm was refined by adapting a previously developed method applicable to the Korean database (Jung *et al.*, 2024; Cho *et al.*, 2026). Detailed methods and relevant code lists are provided in Supplementary File S1 and Supplementary Table S3.

### Potential confounders

For analyses, we adjusted for a comprehensive set of potential confounders, including the following: demographics (e.g. age, insurance type, and calendar year at ET), history of pregnancy (e.g. stillbirth, live birth, induced abortion, ectopic pregnancy, and miscarriage), type of IVF cycle (e.g. conventional IVF, ICSI, and frozen ET [FET]), main indications for benzodiazepine use (e.g. alcohol/substance abuse, tobacco abuse, bipolar disorder, depression/mood disorder, anxiety, sleep disorder, non-affective psychosis, stress-related disorder, eating disorder, personality disorder, and epilepsy/seizures), uterine diseases (e.g. endometrial polyp, endometriosis, pelvic inflammatory disease, uterine myoma, and polycystic ovary syndrome), other comorbidities (e.g. headache/migraine, hypertension, diabetes, renal disease, gastrointestinal disorder, thyroid disorder, and autoimmune disease), medication use (e.g. antidepressants, antipsychotics, Z-drugs, anticonvulsants, and folic acid), and healthcare utilization (e.g. number of outpatient visits, number of hospitalizations, and number of emergency room visits). Details of the potential confounders and their assessment windows are presented in Supplementary Table S3 and Supplementary Fig. S2. In addition to these factors, we also characterized benzodiazepine utilization patterns among users, including route of administration (injection only, oral only, or injection and oral combined), types of agents, and days of supply per prescription.

### Statistical analyses

We first described characteristics of benzodiazepine users versus non-users. For each covariate, we assessed the balance of distribution between the two groups by using the absolute standardized mean difference (aSMD), considering its value of > 0.1 as a significant imbalance. To account for all measured differences, we computed the propensity score (PS) using a multivariable logistic regression model that included all potential confounders listed above. Based on the predicted probability of receiving treatment, overlap weights were calculated to achieve covariate balance between groups (Desai and Franklin, 2019).

To account for the fact that women who fail at an earlier stage of pregnancy are no longer eligible to proceed to subsequent stages, analyses were conducted among the population at risk for each outcome. Accordingly, the clinical pregnancy rate was analyzed among women who reached ET; the miscarriage rate was analyzed among women who achieved clinical pregnancy; and preterm birth and live birth rates were estimated among women who reached 20 weeks of gestation. For each stage-specific analysis, PS was re-estimated, and corresponding weights were applied. For all outcomes, we calculated crude and PS-weighted relative risks (RRs) with 95% CIs using modified Poisson regression models with a log link function, which is the preferred approach to estimate RRs for common outcomes (Chen *et al.*, 2018). To obtain valid standard errors and CIs, we applied a robust variance estimator. All analyses were performed using SAS version 9.4 (SAS Institute Inc., Cary, NC, USA).

### Subgroup and sensitivity analyses

To evaluate the differential impact of benzodiazepine use on outcomes across various subpopulations, we conducted several subgroup analyses. First, to assess the cumulative effects of benzodiazepines, we conducted two subgroup analyses stratified by whether benzodiazepines were prescribed within 3 months before IVF cycle initiation and by cumulative dose. Second, to account for substantial differences in intended use by formulation (Supplementary File S2), analyses were stratified accordingly. Third, to account for effect measure modification by age, considering that advanced female age, particularly beyond 35 years, is associated with reduced fertility (Leridon, 2004), we stratified the analysis by age at the time of ET (< 35, and ≥35 years). Fourth, as the pregnancy rate may differ depending on the fertilization method, we performed subgroup analyses based on the type of fertilization each woman received (conventional IVF, and ICSI). Lastly, we examined whether effect estimates differed by use of other psychiatric medications within 365 days before ET, a proxy for the severity of underlying psychiatric conditions.

We performed multiple sensitivity analyses to examine the robustness of our main findings. First, we employed a 1:1 PS matching method to assess the average treatment effect on treated patients, providing an alternative to the overlap weighting approach. Second, we redefined LMP by subtracting 19 days from the ET date to account for variation in the embryo culture duration. Third, we analyzed without applying a 1-year washout period, thereby including all eligible patients from 1 October 2017, onward. Finally, to account for the potential effects of medications used concomitantly for conscious sedation during IVF procedures, we restricted the analysis to patients who received propofol or opioids, which are commonly administered during oocyte retrieval (Cutting and Metwally, 2022).

### Post hoc analyses

Meanwhile, benzodiazepine may cross the placenta and affect steroidogenesis and cellular proliferation, potentially increasing the risk of preterm birth and miscarriage (Sheehy *et al.*, 2019; Meng *et al.*, 2023, 2024; Li *et al.*, 2026). To minimize potential bias arising from such exposure, we conducted *post hoc* analyses. In the first approach, women in the non-user group who were prescribed benzodiazepine during the expanded exposure window were excluded before estimating the risk, to alleviate potential bias arising from benzodiazepine use during pregnancy among non-users. In the second approach, women who were prescribed benzodiazepine during the expanded exposure window were excluded from both the user and non-user groups to completely eliminate the potential effects of benzodiazepine use after ET on the outcomes (Supplementary Table S4). Additionally, we evaluated the risk of ectopic pregnancy following benzodiazepine use, given that its muscle-relaxant effects on the fallopian tubes may increase the risk of ectopic pregnancy (Wall-Wieler *et al.*, 2020).

## Results

### Study population characteristics

Our study cohort consisted of 99 484 women who completed their first IVF cycle that reached ET. Of these, 28 649 women filled at least one benzodiazepine prescription between LMP and ET, while 61 433 women were not prescribed benzodiazepine within 365 days preceding ET (Fig. 1). Most users received injection type, typically administered 3–5 days before ET, corresponding to the timing of oocyte retrieval. Oral agents were primarily prescribed at the time of ET. Detailed utilization patterns and main indications for benzodiazepine use are presented in Table 1 and Supplementary File S2. When comparing baseline and clinical characteristics between the two groups before PS weighting, we observed substantial differences. Benzodiazepine users showed a lower prevalence of prior live birth (13.4% vs 17.7%) and a higher prevalence of certain psychiatric disorders, along with greater use of antidepressants (anxiety: 8.3% vs 0.4%; antidepressants: 4.3% vs 1.3%). However, covariate balance was achieved after applying PS overlap weights, with all aSMDs < 0.1 (Table 1 and Supplementary Table S5).

**Table 1.** Baseline characteristics of women who completed their first IVF cycle stratified by benzodiazepine use.

	Before overlap weighting		
	Users	Non-users	
Characteristics	(n = 28 649)	(n = 61 433)	aSMD
**Age at ET**			
<25	107 (0.4)	189 (0.3)	0.011
25–29	1733 (6.0)	2958 (4.8)	0.054
30–34	9960 (34.8)	20 422 (33.2)	0.032
35–39	11 551 (40.3)	26 041 (42.4)	0.042
≥40	5298 (18.5)	11 823 (19.2)	0.019
**History of pregnancy**			
Stillbirth	204 (0.7)	425 (0.7)	0.002
Live birth	3842 (13.4)	10 857 (17.7)	**0.118**
Induced abortion	743 (2.6)	1484 (2.4)	0.011
Ectopic pregnancy	908 (3.2)	1784 (2.9)	0.015
Miscarriage	4566 (15.9)	10 177 (16.6)	0.017
**Medical aid recipients**	79 (0.3)	92 (0.1)	0.027
**Calendar year of ET**			
2018	1587 (5.5)	4386 (7.1)	0.033
2019	6316 (22.0)	16 011 (26.1)	0.094
2020	6209 (21.7)	13 749 (22.4)	0.017
2021	6443 (22.5)	12 806 (20.8)	0.040
2022	5648 (19.7)	9860 (16.1)	0.096
2023	2446 (8.5)	4621 (7.5)	0.037
**Type of IVF cycle**			
Conventional IVF	9857 (34.4)	21 729 (35.4)	0.020
ICSI	18 422 (64.3)	34 601 (56.3)	**0.164**
FET	305 (1.1)	4927 (8.0)	**0.339**
Others^a^	65 (0.2)	176 (0.3)	0.012
**Psychiatric conditions**			
Alcohol/substance abuse	46 (0.2)	24 (0.0)	0.038
Tobacco abuse	1 (0.0)	1 (0.0)	0.004
Bipolar disorder	112 (0.4)	50 (0.1)	0.064
Depression/mood disorder	917 (3.2)	408 (0.7)	**0.185**
Anxiety	2381 (8.3)	265 (0.4)	**0.393**
Sleep disorder	705 (2.5)	506 (0.8)	**0.129**
Non-affective psychosis	55 (0.2)	27 (0.0)	0.043
Stress-related disorder	415 (1.4)	302 (0.5)	0.098
Eating disorder	14 (0.0)	10 (0.0)	0.018
Personality disorder	8 (0.0)	5 (0.0)	0.015
Epilepsy/seizures	58 (0.2)	91 (0.1)	0.013
**Uterine diseases**			
Endometrial polyp	3698 (12.9)	7253 (11.8)	0.033
Endometriosis	2355 (8.2)	3823 (6.2)	0.077
Pelvic inflammatory disease	2211 (7.7)	3609 (5.9)	0.073
Uterine myoma	3711 (13.0)	6930 (11.3)	0.051
Polycystic ovary syndrome	3152 (11.0)	8284 (13.5)	0.076
**Comorbidities**			
Headache/migraine	1053 (3.7)	1586 (2.6)	0.063
Hypertension	682 (2.4)	1123 (1.8)	0.039
Diabetes	764 (2.7)	1692 (2.8)	0.005
Renal disease	148 (0.5)	281 (0.5)	0.009
Gastrointestinal disorder	15 352 (53.6)	30 659 (49.9)	0.074
Thyroid disorder	5224 (18.2)	12 964 (21.1)	0.072
Autoimmune disease	3916 (13.7)	7113 (11.6)	0.063
**Comedications**			
Antidepressants	1218 (4.3)	768 (1.3)	**0.184**
Antipsychotics	356 (1.2)	192 (0.3)	**0.106**
Z-drugs	605 (2.1)	505 (0.8)	**0.107**
Anticonvulsants	722 (2.5)	1102 (1.8)	0.050
Folic acid	1275 (4.5)	3592 (5.8)	0.063
**Healthcare utilization**			
Number of outpatient visits, mean (SD)	13.1 (8.6)	11.9 (7.6)	**0.151**
Number of hospitalizations, mean (SD)	0.1 (0.3)	0.0 (0.2)	**0.118**
Number of emergency room visits, mean (SD)	0.1 (0.4)	0.1 (0.3)	0.053
**Benzodiazepine utilization patterns**			
**Route of administration**			
Injection only	23 384 (81.6)	–	–
Oral only	1382 (4.8)	–	–
Injection and oral combined	3883 (13.6)	–	–
**Injectable agents**			
**Type of agents**			
Midazolam	26 332 (91.9)	–	–
Diazepam	955 (3.3)	–	–
Lorazepam	1 (0.0)	–	–
**Oral agents**			
**Type of agents**			
Diazepam	4959 (17.3)	–	–
Alprazolam	203 (0.7)	–	–
Lorazepam	92 (0.3)	–	–
Clonazepam	80 (0.3)	–	–
Flunitrazepam	63 (0.2)	–	–
Triazolam	35 (0.1)	–	–
Bromazepam	22 (0.1)	–	–
Flurazepam	5 (0.0)	–	–
Chlordiazepoxide	3 (0.0)	–	–
Clobazam	1 (0.0)	–	–
**Days of supply per prescription**			
Mean (SD)	2.5 (5.3)	–	–
Median (IQR)	1 (1, 1)	–	–

Bold values indicate absolute standardized mean differences > 0.1. ET, embryo transfer; aSMD, absolute standardized mean difference; FET, frozen embryo transfer.

a Others included cases where two or more cycle types (IVF, ICSI, or FET) were combined within the same cycle.

### IVF outcomes

Before weighting, although a marginally increased risk of preterm birth was observed among users, there were no significant differences between groups in overall outcomes: clinical pregnancy rate (36.0% vs 35.7%; crude RR 1.01 [95% CI 0.99 to 1.03]), miscarriage rate (14.9% vs 15.2%; 0.98 [0.93 to 1.04]), live birth rate (99.3% vs 99.3%; 1.00 [1.00 to 1.00]), and preterm birth rate (13.2% vs 11.6%; 1.15 [1.07 to 1.22]). The results remained similar after adjustment for all potential confounders (clinical pregnancy rate: 1.02 [1.00 to 1.05]; miscarriage rate: 0.99 [0.91 to 1.08]; live birth rate: 1.00 [1.00 to 1.00]; preterm birth rate: 1.10 [0.99 to 1.21]) (Table 2).

**Table 2.** Relative risks for IVF outcomes following benzodiazepine use.

	Users		Non-users		Relative risk (95% CI)	
	No. of events/total^a^	Risk (%)	No. of events/total^a^	Risk (%)	Crude	PS-adjusted
Clinical pregnancy	10 324/28 649	36.0	21 917/61 433	35.7	1.01 (0.99, 1.03)	1.02 (1.00, 1.05)
Miscarriage	1535/10 324	14.9	3329/21 917	15.2	0.98 (0.93, 1.04)	0.99 (0.91, 1.08)
Live birth	8575/8634	99.3	18 107/18 242	99.3	1.00 (1.00, 1.00)	1.00 (1.00, 1.00)
Preterm birth	1150/8634	13.3	2122/18 242	11.6	1.15 (1.07, 1.22)	1.10 (0.99, 1.21)

PS, propensity score.

a Denominator for each outcome: to account for the fact that women who fail at an earlier stage of pregnancy are no longer eligible to proceed to subsequent stages, analyses were conducted among the population at risk for each outcome. Accordingly, clinical pregnancy rate was analyzed among women who reached ET; miscarriage rate was analyzed among women who achieved clinical pregnancy; and live birth and preterm birth rates were estimated among women who reached 20 weeks of gestation.

### Subgroup, sensitivity, and post hoc analyses

Subgroup and sensitivity analyses yielded results generally consistent with the primary findings (Tables 3 and 4, and Supplementary Table S6). However, in some subgroups, the risk of preterm birth was higher than that observed in the main analysis. When analyses were restricted to women with benzodiazepine prescriptions within 3 months prior to IVF initiation, the point estimate for preterm birth was 1.21 (0.79 to 1.85) (Table 3). Among women undergoing ICSI or those without other psychiatric medication use during the year before ET, benzodiazepine users had a significantly higher risk of preterm birth (1.16 [1.02 to 1.32] and 1.10 [1.00 to 1.22], respectively) (Table 4).

**Table 3.** Subgroup analyses of relative risks for IVF outcomes following benzodiazepine use stratified by history of prescription, cumulative dose, and type of formulation.

	Users		Non-users		Relative risk (95% CI)	
	No. of events/total^a^	Risk (%)	No. of events/total^a^	Risk (%)	Crude	PS-adjusted
**Clinical pregnancy**						
Total	10 324/28 649	36.0	21 917/61 433	35.7	1.01 (0.99, 1.03)	1.02 (1.00, 1.05)
History of benzodiazepine prescription^b^						
Yes	354/1118	31.7	21 917/61 433	35.7	0.89 (0.81, 0.97)	1.00 (0.90, 1.11)
No	9491/26 136	36.3	21 917/61 433	35.7	1.02 (1.00, 1.04)	1.05 (1.01, 1.09)
Cumulative dose^c^						
≤1.2 mg	3804/9087	41.9	21 917/61 433	35.7	1.17 (1.14, 1.20)	1.18 (1.15, 1.21)
1.2 to 2 mg	4361/13 092	33.3	21 917/61 433	35.7	0.93 (0.91, 0.96)	0.92 (0.90, 0.95)
>2 mg	2159/6470	33.4	21 917/61 433	35.7	0.94 (0.90, 0.97)	1.01 (0.97, 1.05)
Type of formulation						
Injection only	8606/23 384	36.8	21 917/61 433	35.7	1.03 (1.01, 1.05)	1.03 (1.00, 1.06)
Oral only	484/1382	35.0	21 917/61 433	35.7	0.98 (0.91, 1.06)	1.01 (0.89, 1.14)
Injection + oral	1234/3883	31.8	21 917/61 433	35.7	0.89 (0.85, 0.93)	1.02 (0.95, 1.10)
**Miscarriage**						
Total	1535/10 324	14.9	3329/21 917	15.2	0.98 (0.93, 1.04)	0.99 (0.91, 1.08)
History of benzodiazepine prescription^b^						
Yes	71/354	20.5	3329/21 917	15.2	1.32 (1.07, 1.63)	0.97 (0.62, 1.51)
No	1397/9491	14.7	3329/21 917	15.2	0.97 (0.91, 1.03)	0.99 (0.94, 1.05)
Cumulative dose^c^						
≤1.2 mg	567/3804	14.9	3329/21 917	15.2	0.98 (0.90, 1.07)	0.97 (0.90, 1.06)
1.2 to 2 mg	663/4361	15.2	3329/21 917	15.2	1.00 (0.93, 1.08)	1.05 (0.97, 1.13)
>2 mg	305/2159	14.1	3329/21 917	15.2	0.93 (0.83, 1.04)	0.90 (0.79, 1.02)
Type of formulation						
Injection only	1301/8606	15.1	3329/21 917	15.2	1.00 (0.94, 1.06)	1.02 (0.94, 1.11)
Oral only	75/484	15.5	3329/21 917	15.2	1.02 (0.83, 1.26)	0.87 (0.60, 1.25)
Injection + oral	159/1234	12.9	3329/21 917	15.2	0.85 (0.73, 0.98)	0.83 (0.65, 1.07)
**Live birth**						
Total	8575/8634	99.3	18 107/18 242	99.3	1.00 (1.00, 1.00)	1.00 (1.00, 1.00)
History of benzodiazepine prescription^b^						
Yes	273/274	99.6	18 107/18 242	99.3	1.00 (1.00, 1.01)	1.00 (1.00, 1.01)
No	7899/7955	99.3	18 107/18 242	99.3	1.00 (1.00, 1.00)	1.00 (1.00, 1.00)
Cumulative dose^c^						
≤1.2 mg	3178/3198	99.4	18 107/18 242	99.3	1.00 (1.00, 1.00)	1.00 (1.00, 1.00)
1.2 to 2 mg	3585/3609	99.3	18 107/18 242	99.3	1.00 (1.00, 1.00)	1.00 (1.00, 1.00)
>2 mg	1812/1827	99.2	18 107/18 242	99.3	1.00 (0.99, 1.00)	1.00 (0.99, 1.00)
Type of formulation						
Injection only	7128/7173	99.4	18 107/18 242	99.3	1.00 (1.00, 1.00)	1.00 (1.00, 1.00)
Oral only	397/400	99.3	18 107/18 242	99.3	1.00 (0.99, 1.01)	0.99 (0.99, 1.00)
Injection + oral	1050/1061	99.0	18 107/18 242	99.3	1.00 (0.99, 1.00)	1.00 (0.99, 1.00)
**Preterm birth**						
Total	1150/8634	13.3	2122/18 242	11.6	1.15 (1.07, 1.22)	1.10 (0.99, 1.21)
History of benzodiazepine prescription^b^						
Yes	40/274	14.6	2122/18 242	11.6	1.26 (0.94, 1.68)	1.21 (0.79, 1.85)
No	1056/7955	13.3	2122/18 242	11.6	1.14 (1.07, 1.22)	1.10 (1.02, 1.18)
Cumulative dose^c^						
≤1.2 mg	421/3198	13.2	2122/18 242	11.6	1.13 (1.03, 1.25)	1.09 (0.99, 1.21)
1.2 to 2 mg	502/3609	13.9	2122/18 242	11.6	1.20 (1.09, 1.31)	1.13 (1.03, 1.24)
>2 mg	227/1827	12.4	2122/18 242	11.6	1.07 (0.94, 1.21)	1.01 (0.87, 1.17)
Type of formulation						
Injection only	973/7173	13.6	2122/18 242	11.6	1.17 (1.09, 1.25)	1.11 (1.03, 1.19)
Oral only	47/400	11.8	2122/18 242	11.6	1.01 (0.77, 1.33)	1.09 (0.69, 1.74)
Injection + oral	130/1061	12.3	2122/18 242	11.6	1.05 (0.89, 1.24)	1.04 (0.86, 1.26)

ET, embryo transfer; PS, propensity score.

a Denominator for each outcome: to account for the fact that women who fail at an earlier stage of pregnancy are no longer eligible to proceed to subsequent stages, analyses were conducted among the population at risk for each outcome. Accordingly, clinical pregnancy rate was analyzed among women who reached ET; miscarriage rate was analyzed among women who achieved clinical pregnancy; and preterm birth and live birth rates were estimated among women who reached 20 weeks of gestation.

b To account for potential residual effects of prior benzodiazepine exposure, users were stratified according to whether they had received a benzodiazepine prescription during the 90 days before LMP (But we excluded individuals who used benzodiazepine for infertility within 90 days before LMP to minimize potential confounding related to fertility. The indication for benzodiazepine prescriptions was identified using diagnosis information recorded on the same billing statement. In the Korean claims system, only reimbursed indications approved by the National Health Insurance Service are recorded, while non-reimbursed uses are not captured. Therefore, the diagnosis information included in the prescription claims reflects the indication for medication use).

c We calculated the cumulative dose in diazepam equivalents (Supplementary Table S2). The cumulative dose categories were defined based on both dose distribution and tertiles.

**Table 4.** Subgroup analyses of relative risks for IVF outcomes following benzodiazepine use stratified by patients’ characteristics.

	Users		Non-users		Relative risk (95% CI)	
	No. of events/total^a^	Risk (%)	No. of events/total^a^	Risk (%)	Crude	PS-adjusted
**Clinical pregnancy**						
Total	10 324/28 649	36.0	21 917/61 433	35.7	1.01 (0.99, 1.03)	1.02 (1.00, 1.05)
Age group						
<35	5088/11 800	43.1	10 122/23 569	43.0	1.00 (0.98, 1.03)	1.01 (0.97, 1.05)
≥35	5236/16 849	31.1	11 795/37 864	31.2	1.00 (0.97, 1.02)	1.04 (1.00, 1.08)
Type of fertilization procedure						
Conventional IVF	4016/9857	40.7	8482/21 729	39.0	1.04 (1.01, 1.07)	1.04 (1.00, 1.09)
ICSI	6179/18 422	33.5	11 504/34 601	33.3	1.01 (0.98, 1.03)	1.01 (0.98, 1.05)
Other psychiatric medications use						
Yes	695/2116	32.8	787/2312	34.0	0.96 (0.89, 1.05)	1.00 (0.87, 1.14)
No	9629/26 533	36.3	21 130/59 121	35.7	1.02 (1.00, 1.04)	1.03 (1.00, 1.06)
**Miscarriage**						
Total	1535/10 324	14.9	3329/21 917	15.2	0.98 (0.93, 1.04)	0.99 (0.91, 1.08)
Age group						
<35	501/5088	9.9	1013/10 122	10.0	0.98 (0.89, 1.09)	1.00 (0.86, 1.16)
≥35	1034/5236	19.8	2316/11 795	19.6	1.01 (0.94, 1.07)	0.99 (0.90, 1.09)
Type of fertilization procedure						
Conventional IVF	603/4016	15.0	1156/8482	13.6	1.10 (1.01, 1.21)	1.10 (0.96, 1.26)
ICSI	907/6179	14.7	1849/11 504	16.1	0.91 (0.85, 0.98)	0.92 (0.83, 1.02)
Other psychiatric medications use						
Yes	125/695	18.0	122/787	15.5	1.16 (0.92, 1.46)	1.11 (0.77, 1.62)
No	1410/9629	14.6	3207/21 130	15.2	0.96 (0.91, 1.02)	0.98 (0.90, 1.07)
**Live birth**						
Total	8575/8634	99.3	18 107/18 242	99.3	1.00 (1.00, 1.00)	1.00 (1.00, 1.00)
Age group						
<35	4505/4531	99.4	8917/8984	99.3	1.00 (1.00, 1.00)	1.00 (1.00, 1.01)
≥35	4070/4103	99.2	9190/9258	99.3	1.00 (1.00, 1.00)	1.00 (1.00, 1.00)
Type of fertilization procedure						
Conventional IVF	3335/3353	99.5	7131/7184	99.3	1.00 (1.00, 1.01)	1.00 (1.00, 1.01)
ICSI	5138/5179	99.2	9418/9491	99.2	1.00 (1.00, 1.00)	1.00 (1.00, 1.00)
Other psychiatric medications use						
Yes	543/554	98.0	651/656	99.2	0.99 (0.97, 1.00)	0.99 (0.97, 1.01)
No	8032/8080	99.4	17 456/17 586	99.3	1.00 (1.00, 1.00)	1.00 (1.00, 1.00)
**Preterm birth**						
Total	1150/8634	13.3	2122/18 242	11.6	1.15 (1.07, 1.22)	1.10 (0.99, 1.21)
Age group						
<35	618/4531	13.6	1025/8984	11.4	1.20 (1.09, 1.31)	1.13 (0.98, 1.30)
≥35	532/4103	13.0	1097/9258	11.9	1.10 (0.99, 1.21)	1.06 (0.92, 1.23)
Type of fertilization procedure						
Conventional IVF	444/3353	13.2	910/7184	12.7	1.05 (0.94, 1.16)	1.01 (0.86, 1.18)
ICSI	697/5179	13.5	1062/9491	11.2	1.20 (1.10, 1.32)	1.16 (1.02, 1.32)
Other psychiatric medications use						
Yes	87/554	15.7	100/656	15.2	1.03 (0.79, 1.34)	0.93 (0.60, 1.45)
No	1063/8080	13.2	2022/17 586	11.5	1.14 (1.07, 1.23)	1.10 (1.00, 1.22)

ET, embryo transfer; PS, propensity score.

a Denominator for each outcome: to account for the fact that women who fail at an earlier stage of pregnancy are no longer eligible to proceed to subsequent stages, analyses were conducted among the population at risk for each outcome. Accordingly, clinical pregnancy rate was analyzed among women who reached ET; miscarriage rate was analyzed among women who achieved clinical pregnancy; and preterm birth and live birth rates were estimated among women who reached 20 weeks of gestation.

In contrast, stratified analyses by cumulative benzodiazepine dose yielded results similar to the main analysis (≤1.2 mg and >2 mg, respectively—clinical pregnancy: 1.18 [1.15 to 1.21] and 1.01 [0.97 to 1.05]; miscarriage: 0.97 [0.90 to 1.06] and 0.90 [0.79 to 1.02]; live birth: 1.00 [1.00 to 1.00] and 1.00 [0.99 to 1.00]; preterm birth: 1.09 [0.99 to 1.21] and 1.01 [0.87 to 1.17]). Moreover, among users prescribed both injectable and oral agents during the cycle, the estimated effect was closer to the null (preterm birth: injection only, 1.11 [1.03 to 1.19]; oral only, 1.09 [0.69 to 1.74]; injection and oral combined, 1.04 [0.86 to 1.26]) (Table 3). Additional analyses accounting for benzodiazepine use during pregnancy yielded results similar to those of the main analysis (Supplementary Table S4). Ectopic pregnancy rates were comparable between groups (0.94 [0.83 to 1.08]) (Supplementary Table S7).

## Discussion

In this cohort study of 99 484 women undergoing IVF—including 28 649 who were prescribed benzodiazepines throughout the IVF cycle—we found that benzodiazepine use was not associated with meaningful differences in pregnancy outcomes. Although a marginal increase in preterm birth risk was observed, no dose–response relationship was identified, and women prescribed both oral and injectable formulations within the same cycle did not exhibit a higher risk. Overall, these results suggest that benzodiazepine exposure is unlikely to substantially affect pregnancy outcomes, and they were consistent across comprehensive subgroup, sensitivity, and supplementary analyses.

Our findings are generally concordant with previous studies evaluating the safety or effectiveness of benzodiazepine use at specific time points, such as oocyte retrieval or ET, on reproductive outcomes. In the ART setting, benzodiazepines can be used for a variety of purposes. First, as agents for conscious sedation, several animal studies have demonstrated that short-term benzodiazepine use does not adversely affect IVF outcomes (Swanson and Leavitt, 1992; Van Wemmel *et al.*, 2005). Based on this evidence, benzodiazepines, specifically midazolam, have been widely used during oocyte retrieval since 1990s (Trout *et al.*, 1998). These safety profiles were similarly identified in the present study. Second, in addition to their sedative properties, benzodiazepines have been suggested to improve ET outcomes (Koga *et al.*, 1992; Duggan *et al.*, 2002), and a single-center study supported this hypothesis by reporting a higher clinical pregnancy rate among women who received oral diazepam before ET (66.2% vs 62.1%, *P* < 0.03) (Kalluru *et al.*, 2021). However, a subsequent multicenter study using data from 12 fertility clinics in Portugal and Spain found no significant differences between oral diazepam users and non-users in clinical pregnancy rate (45.6% vs 46.2%, adjusted *P* = 0.11), miscarriage rate (11.0% vs 9.3%, adjusted *P* = 0.26), live birth rate (36.3% vs 35.3%, adjusted *P* = 0.82), or preterm birth rate (0.3% vs 0.0%, adjusted *P* = 0.99), suggesting limited effectiveness in enhancing treatment success (Brandão *et al.*, 2024). Consistent with the findings of this most recent study, we observed no beneficial effects of benzodiazepine use on pregnancy outcomes, including in formulation-specific analyses. Furthermore, unlike previous studies limited to specific procedural time points, the present study evaluated benzodiazepine exposure across the entire IVF cycle from LMP to ET and comprehensively adjusted for a broad set of potential confounders not adequately addressed in prior research, thereby providing more robust evidence regarding the association between benzodiazepine use and reproductive outcomes.

While broadly reassuring, our findings also merit closer attention to a small increase in preterm birth risk observed in certain subgroups, sensitivity, and *post hoc* analyses, and several plausible mechanisms may underlie this finding. Notably, an ∼10% higher risk remained in the analysis restricted to women who had not been prescribed benzodiazepines within 3 months before IVF cycle initiation. One possible explanation is that residual effects of benzodiazepine exposure around the time of conception may have adversely influenced fetal development and increased the risk of preterm birth. Benzodiazepines can cross the placental barrier and accumulate in embryonic and fetal tissues, and modulation of the γ-aminobutyric acid (GABA) receptor has been implicated in abnormalities of early placental development and fetal growth (Meng *et al.*, 2023). More specifically, benzodiazepines may act on GABA-A receptors expressed in the endometrium and early trophoblast, thereby impairing trophoblast invasion and spiral artery remodeling during early placentation, processes that have been implicated in the pathogenesis of preterm birth (Sadeghi and Taylor, 2010; Lu *et al.*, 2016; Varberg and Soares, 2021). Furthermore, in the subgroup of women who had not used any psychiatric medications for at least 1 year before ET—a population less likely to be affected by unmeasured confounding related to psychiatric disease severity—the association remained comparable to that observed in the main analysis. Therefore, the potential association between benzodiazepine use and preterm birth cannot be completely ruled out, and the observed increase in risk among women with specific maternal characteristics should be interpreted with caution.

Nevertheless, the observed effect size was small and was not consistently replicated across analyses. Moreover, residual confounding arising from underlying patient characteristics may also have contributed to the observed association. Taken together, our findings support the overall safety of benzodiazepine use in women undergoing IVF. However, individualized risk-benefit assessment remains warranted, and further studies in diverse populations, incorporating more detailed information on baseline obstetric and clinical characteristics, are needed to clarify this relationship and improve the generalizability of these findings.

### Strengths and limitations

In South Korea, all infertile couples have been eligible for government subsidies for ART, enabling us to capture a large population-based cohort that encompasses most ART cycles nationwide. As each step of the ART process and prescribing indications must be accurately recorded to receive reimbursement, we were able to define precise assessment windows anchored to the date of ET and accurately identify the major indications and timing of benzodiazepine prescriptions.

As with any retrospective study based on administrative data, this study also has inherent limitations. First, some women may have undergone ART procedures before the study period, as infertility treatment was not government-covered until October 2017, limiting our ability to capture complete ART histories. Although we implemented a 1-year washout period to increase the likelihood of capturing true first cycles, the possibility of selection bias remains. Second, prescription presence does not guarantee actual medication use, potentially leading to exposure misclassification. Women planning pregnancy may be more cautious about medication use, resulting in differential adherence patterns that could either underestimate or overestimate associations. Third, this database lacks direct information on pregnancy outcomes and IVF-related outcomes. To address this limitation, we constructed a hierarchical algorithm to identify pregnancy episodes based on a previously developed approach and defined clinical pregnancy, and other IVF outcomes accordingly. All pregnancy episodes were defined using diagnosis and procedure codes within a prespecified relevant time window. These codes were recorded for reimbursement purposes, which involve financial implications, and the definitions were established through clinician consultation, suggesting a relatively high level of accuracy. Nevertheless, the possibility of outcome misclassification bias cannot be entirely excluded. Lastly, confounding by unmeasured variables such as disease severity, genetic, or environmental factors cannot be entirely ruled out. Despite controlling for extensive potential confounders and conducting multiple sensitivity analyses, residual confounding may persist and results should be interpreted cautiously.

## Conclusions

Benzodiazepine use during the IVF cycle was not associated with overall pregnancy outcomes, except for a marginal increase in the risk of preterm birth. However, a potential association cannot be fully ruled out. Therefore, careful consideration of patients’ baseline obstetric and clinical characteristics is essential when determining benzodiazepine use. Further studies in diverse populations are needed to better define its safety and effectiveness and to support its use as a treatment option for pain relief and psychological stabilization throughout the IVF cycle.

## Supplementary Material

hoag065_Supplementary_Data

## Data Availability

The data cannot be shared publicly due to restrictions imposed by the data-sharing policy of the Health Insurance Review and Assessment Service (HIRA) of Korea. This study was approved by the Institutional Review Board of Sungkyunkwan University, South Korea (No. 2025-06-052).
