# Supplementary material for: Benzodiazepine use during an IVF cycle and its impacts on miscarriage and other pregnancy outcomes
Source: Hum Reprod Open. 2026 Jul 17;2026(3):hoag065. doi: 10.1093/hropen/hoag065 (PMC13441166; doi:10.1093/hropen/hoag065)
Supplement: Supplementary file 1 [file hoag065_supplementary_data.docx]

**Supplemental Online Content**

**Supplementary Figure S1.** Study period

**Supplementary Figure S2.** Study design diagram

**Supplementary Table S1.** Protocol of a target trial

**Supplementary Table S2.** Benzodiazepines category and ATC codes

**Supplementary Table S3.** Codes used to define inclusion criteria, outcomes, and covariates

**Supplementary Table S4.** Supplementary analyses with consideration of benzodiazepine exposure during pregnancy

**Supplementary Table S5.** Baseline characteristics of women who completed their first IVF cycle stratified by benzodiazepine use after overlap weighting

**Supplementary Table S6.** Sensitivity analyses

**Supplementary Table S7.** Supplementary analysis for ectopic pregnancy rate

**Supplementary File S1.** Definition of pregnancy outcomes after IVF cycle

**Supplementary File S2.** Utilization trend of benzodiazepine for infertile women undergoing IVF cycle

# **Supplementary Figure S1.** Study period

# **Supplementary Figure S2.** Study design diagram

**Abbreviations:** ET, embryo transfer; LMP, last menstrual period

^a^ To identify the first IVF cycle that progressed to ET, we included only cycles that involved IVF, ICSI, or thawing procedures followed by ET within 5 days, in alignment with the standard IVF cycle schedule.

^b^ LMP date was estimated by subtracting 17 days from the ET date.

**Supplementary Table S1.** **Protocol of a target trial**

| Protocol Component | Target trial | Emulation |
| --- | --- | --- |
| Eligibility criteria | Inclusion criteria:  Women who plan to conduct their first IVF cycle. | Same as the target trial, except that we restricted to women who reached to embryo transfer (ET). We assumed that cycles that did not reach ET may have been primarily affected by male factor infertility. |
| Treatment strategies | 1. Users: benzodiazepine use during an IVF cycle 2. Non-users: no benzodiazepine use during an IVF cycle | Same as the target trial,  Operationalized as:   1. Users: ≥ 1 dispensation of benzodiazepine between the last menstrual period (LMP) to ET 2. Non-users: No dispensation of benzodiazepine between LMP to ET (to minimize exposure misclassification, the non-user group was restricted to individuals with no benzodiazepine prescriptions between 365 and 18 days prior to ET)   The LMP was estimated by subtracting 17 days from the ET date |
| Treatment assignment | Participants would be randomly assigned to one of the two treatment strategies and would be aware of their assignment. | We assumed randomization within levels of the following baseline covariates: age, insurance type, calendar year, pregnancy history, type of fertilization, psychiatric conditions, other comorbidities, comedications, and health utilization |
| Outcomes | 1. Clinical pregnancy rate 2. Miscarriage rate 3. Live birth rate 4. Preterm birth rate | Same as the target trial  These outcomes were defined based on pregnancy outcomes following ET and were identified using a hierarchical algorithm based on relevant time windows and procedure or diagnosis codes.  Details are provided in **Supplementary File S1.** |
| Follow-up | Follow-up begins at randomization (LMP) and continues until the outcome of interest or the end of pregnancy, whichever occurred first. | Follow-up began at the end of treatment assignment window (ET) and continued until the outcome of interest or the end of pregnancy, whichever occurred first. |
| Causal contrasts | Intention-to-treat effect | Observational analog of the intention-to-treat effect |
| Statistical analysis | Relative risks (RRs) with 95% confidence intervals (CIs) are estimated using Poisson regression in the selected study population. | Same as the target trial, except that we adjusted for confoundings additionally.  RRs with 95% CIs were estimated using Poisson regression in an overlap-weighted at-risk population cohort for each outcome |

# **Supplementary Table S2.** **Benzodiazepines category and ATC codes**

| **Agents** | **ATC codes** | **Equivalence dose (mg)^a^** | |
| --- | --- | --- | --- |
|  |  | **Oral^1^** | **Injection^2^** |
| Alprazolam | N05BA12 | 0.5 | - |
| Lorazepam | N05BA06 | 1 | 1 |
| Midazolam | N05CD08 | - | 2 |
| Triazolam | N05CD05 | 0.5 | - |
| Flurazepam | N05CD01 | 15-30 | - |
| Clobazam | N05BA09 | 20 | - |
| Clonazepam | N03AE01 | 0.5 | - |
| Diazepam | N05BA01 | 10 | 5 |
| Flunitrazepam | N05CD03 | 1 | - |
| Chlordiazepoxide | N05BA02 | 25 | - |
| Bromazepam | N05BA08 | 5-6 | - |

^a^ The approximate equivalent doses to diazepam are given.

**Abbreviations:** ATC, Anatomical Therapeutic Classification

^1^ <https://www.scribd.com/document/72899196/Benzodiazepine-Equivalents-Table> (access date: Mar 16, 2026)

^2^ <https://www.ciusss-capitalenationale.gouv.qc.ca/en/antidotes/benzodiazepines> (access date: Mar 16, 2026)

# **Supplementary Table S3. Codes used to define inclusion criteria, outcomes, and covariates^a^**

| **Categories** | **Codes^b^** | **Details** |
| --- | --- | --- |
| **Inclusion Criteria** |  |  |
| Female infertility | Dx | N97 |
| Ovulation induction drugs | Rx | G03GA, G03GB02, L02BG04, L02AE, H01CC |
| Oocyte retrieval | Px | R6420, R6430, R6431, R6432, R6440, R6441, R6442, R6450, R6451, R6452, R6460, R6461, R6462 |
| Embryo transfer | Px | R6532, R6533, R6530, R6531, R6540, R6550 |
| **Pregnancy outcomes** |  |  |
| Clinical pregnancy | Px | EB511, EB512 |
| Miscarriage | Px | R4441, R4442 |
|  | Dx | O02-O06 (excl.O04) |
| Live birth / Preterm birth | Px | R3131, R3136, R3141, R3146, R4351, R4356, R4361, R4362, R4514, R4517, R4518, R4507, R4508, R4509, R4510, R4380, RA361, RA362, RA380, RA361, RA362, RA380, RA311, RA313, RA315, RA317, RA431, RA433, R3133, R3138, R3143, R3148, R4353, R4358, R4516, R4519, R4520, R5001, R5002, RA312, RA314, RA316, RA318, RA432, RA434 |
| Stillbirth | Px | F4459, F4460 |
|  | Dx | Z3710, Z3711, Z3712, Z3713, Z3740, Z3741, Z3742, Z3743, Z3770, Z3771, Z3772, Z3773, P95, Z371, Z374, Z377, O364 |
| **Competing events** |  |  |
| Induced abortion | Px | R4452, R4453, R4456, R4457, R4458, RY541, RY542, RY543, RY544 |
|  | Dx | O04 |
| Ectopic pregnancy | Px | R4531, R4532, R4533, R4534 |
|  | Dx | O000, O001, O002, O008 |
| **Psychiatric conditions^c^** |  |  |
| Alcohol/substance abuse | Dx | F10-F19 (excl. F17), Z714, Z715, Z721, Z722 |
| Tobacco abuse | Dx | F17, Z716, Z720 |
| Bipolar disorder | Dx | F30, F31 |
| Depression/mood disorder | Dx | F32-F34, F38, F39, F412 |
| Anxiety | Dx | F40, F41 |
| Sleep disorder | Dx | F51, G47 |
| Non-affective psychosis | Dx | F20-F29 |
| Stress-related disorder | Dx | F42-F48 |
| Eating disorder | Dx | F50 |
| Personality disorder | Dx | F60-F63, F68-F69 |
| Epilepsy/seizures | Dx | G40, G41 |
| **Uterine diseases^c^** |  |  |
| Endometrial polyp | Dx | N84 |
| Endometriosis | Dx | N80 |
| Pelvic inflammatory disease | Dx | N73 |
| Uterine myoma | Dx | D25 |
| Polycystic ovary syndrome | Dx | E282 |
| **Comorbidities^c^** |  |  |
| Headache/migraine | Dx | G43, G44 |
| Hypertension | Dx | I10-I15 (excl. I14) |
| Diabetes | Dx | E10-E14 |
| Renal disease | Dx | E112, E132, E142, I12, I13, N00-N08, N17-N19, N25-N27 |
| Gastrointestinal disorder | Dx | K20, K21, K25-K29, K58 |
| Thyroid disorder | Dx | E01-E07 (excl. E04) |
| Autoimmune disease | Dx | B20-B24, D80-D89 (excl. D85, D87), M05, M06, M30-M34, M351, M353, M45, M79, K50, K51, G35, G700, L40 |
| **Comedications^c^** |  |  |
| Antidepressants | Rx | N06A (mirtazapine, moclobemide, nefazodone, tianeptine, trazodone, bupropion, agomelatine, vortioxetine, milnacipran, duloxetine, desvenlafaxine, fluoxetine, fluvoxamine, paroxetine, sertraline, citalopram, escitalopram, amitriptyline, amoxapine, clomipramine, doxepin, imipramine, maprotiline, quinupramine) |
| Antipsychotics | Rx | N05A (quetiapine, olanzapine, clozapine, flupentixol, zuclopenthixol, chlorprothixene, prochlorperazine, thioridazine, perphenazine, levomepromazine, chlorpromazine, paliperidone, aripiprazole, risperidone, ziprasidone, pimozide, melperone, haloperidol, haloperidol, amisulpride, sulpiride, tiapride) |
| Z-drugs | Rx | N05CF (eszopiclone, zolpidem, zopiclone) |
| Anticonvulsants | Rx | N03 (levetiracetam, pregabalin, phenytoin, valproate, topiramate, lamotrigine, gabapentin, phenytoin, carbamazepine) |
| Folic acid | Rx | B03BB |

**Abbreviations:** Dx, diagnosis; Rx, prescription; Px, procedure

^a^ Demographic characteristics (e.g., age, insurance type, and calendar year at embryo transfer [ET]) were assessed as of the date of ET; Parity was determined based on the presence of any delivery procedure code prior to ET; The type of fertilization was identified within the 5 days preceding ET; Healthcare utilization was measured during the 180-day period prior to the date of ET.

^b^ Prescription and diagnosis codes are based on Anatomical Therapeutic Classification (ATC) and International Classification of Diseases 10th revision (ICD-10) classification systems, respectively. For procedures, the domestic coding systems used in South Korea were applied.

^c^ Psychiatric conditions, uterine diseases, comorbidities, and comedications were measured during 1-year period before the date of ET.

# **Supplementary Table S4. Supplementary analyses with consideration of benzodiazepine exposure during pregnancy**

| (1) **Excluding women who were prescribed benzodiazepines during pregnancy from non-user group** | | | | | | |
| --- | --- | --- | --- | --- | --- | --- |
|  | **Users** | | **Non-users** | | **Relative risk (95% CI)** | |
|  | **No. of Events/total** | **Risk (%)** | **No. of Events/total** | **Risk (%)** | **Crude** | **PS-adjusted** |
| Miscarriage | 1535/10 324 | 14.9 | 3327/21 910 | 15.2 | 0.98 (0.93, 1.04) | 0.99 (0.93, 1.05) |
| Live birth | 8575/8634 | 99.3 | 18 054/18 178 | 99.3 | 1.00 (1.00, 1.00) | 1.00 (1.00, 1.00) |
| Preterm birth | 1150/8634 | 13.3 | 2108/18 178 | 11.6 | 1..15 (1.07, 1.23) | 1.10 (1.02, 1.18) |

**Abbreviations:** PS, propensity score

| (2) **Excluding women who were prescribed benzodiazepines during pregnancy from both user and non-user group** | | | | | | |
| --- | --- | --- | --- | --- | --- | --- |
|  | **Users** | | **Non-users** | | **Relative risk (95% CI)** | |
|  | **No. of Events/total** | **Risk (%)** | **No. of Events/total** | **Risk (%)** | **Crude** | **PS-adjusted** |
| Miscarriage | 1528/10 293 | 14.9 | 3327/21 910 | 15.2 | 0.98 (0.92, 1.03) | 0.99 (0.93, 1.05) |
| Live birth | 8504/8557 | 99.4 | 18 054/18 178 | 99.3 | 1.00 (1.00, 1.00) | 1.00 (1.00, 1.00) |
| Preterm birth | 1133/8557 | 13.2 | 2108/18 178 | 11.6 | 1.14 (1.07, 1.22) | 1.09 (1.02, 1.17) |

**Abbreviations:** PS, propensity score

- For considering effect of exposure during pregnancy, we conducted two additional supplementary analyses.

  (1) To address potential bias related to benzodiazepine use during pregnancy, we excluded women in the non-user group who were prescribed benzodiazepines during the expanded exposure window.
  (2) To completely exclude the effects of benzodiazepine use after pregnancy onset on the outcomes, we excluded women who were prescribed benzodiazepines during the expanded exposure window from both the user and non-user groups.

**Expanded exposure assessment window during pregnancy:**

(1) Miscarriage: date of embryo transfer + 1 day to + 14 days
(2) Livebirth/preterm birth: date of embryo transfer + 1 day to + 140 days

Given that the first sonography typically occurs 2–3 weeks after embryo transfer, the exposure window was extended by 14 days to estimate the risk of miscarriage among women who achieved a clinical pregnancy.
For the analyses of live birth and preterm birth rates, the exposure window was extended by 140 days, as delivery occurs after 20 gestational weeks (140 days).

# **Supplementary Table S5. Baseline characteristics of women who completed their first IVF cycle stratified by benzodiazepine use after overlap weighting**

|  |  | **After overlap weighting** | | |
| --- | --- | --- | --- | --- |
|  |  | **Users** | **Non-users** |  |
| **Characteristics** |  | **(n=17 880)** | **(n=17 880)** | **aSMD** |
| **Age at ET** |  |  |  |  |
| <25 |  | 65 (0.4) | 65 (0.4) | 0.000 |
| 25-29 |  | 1024 (5.7) | 1024 (5.7) | 0.000 |
| 30-34 |  | 6189 (34.6) | 6189 (34.6) | 0.000 |
| 35-39 |  | 7291 (40.8) | 7291 (40.8) | 0.000 |
| ≥40 |  | 3311 (18.5) | 3311 (18.5) | 0.000 |
| **History of pregnancy** |  |  |  |  |
| Stillbirth |  | 121 (0.7) | 121 (0.7) | 0.000 |
| Live birth |  | 2429 (13.6) | 2429 (13.6) | 0.000 |
| Induced abortion |  | 446 (2.5) | 446 (2.5) | 0.000 |
| Ectopic pregnancy |  | 533 (3.0) | 533 (3.0) | 0.000 |
| Miscarriage |  | 2835 (15.9) | 2835 (15.9) | 0.000 |
| **Medical aid recipients** |  | 36 (0.2) | 36 (0.2) | 0.000 |
| **Calendar year of ET** |  |  |  |  |
| 2018 |  | 1033 (5.8) | 1033 (5.8) | 0.000 |
| 2019 |  | 4119 (23.0) | 4119 (23.0) | 0.000 |
| 2020 |  | 3931 (22.0) | 3931 (22.0) | 0.000 |
| 2021 |  | 3983 (22.3) | 3983 (22.3) | 0.000 |
| 2022 |  | 3327 (18.6) | 3327 (18.6) | 0.000 |
| 2023 |  | 1487 (8.3) | 1487 (8.3) | 0.000 |
| **Type of fertilization** |  |  |  |  |
| Conventional IVF |  | 6245 (34.9) | 6,245 (34.9) | 0.000 |
| ICSI |  | 11 363 (63.6) | 11 363 (63.6) | 0.000 |
| FET |  | 231 (1.3) | 231 (1.3) | 0.000 |
| Others^a^ |  | 40 (0.2) | 40 (0.2) | 0.000 |
| **Psychiatric conditions** |  |  |  |  |
| Alcohol/substance abuse |  | 13 (0.1) | 13 (0.1) | 0.000 |
| Tobacco abuse |  | 0 (0.0) | 0 (0.0) | 0.000 |
| Bipolar disorder |  | 27 (0.2) | 27 (0.2) | 0.000 |
| Depression/mood disorder |  | 236 (1.3) | 236 (1.3) | 0.000 |
| Anxiety |  | 241 (1.3) | 241 (1.3) | 0.000 |
| Sleep disorder |  | 239 (1.3) | 239 (1.3) | 0.000 |
| Non-affective psychosis |  | 16 (0.1) | 16 (0.1) | 0.000 |
| Stress-related disorder |  | 145 (0.8) | 145 (0.8) | 0.000 |
| Eating disorder |  | 4 (0.0) | 4 (0.0) | 0.000 |
| Personality disorder |  | 2 (0.0) | 2 (0.0) | 0.000 |
| Epilepsy/seizures |  | 30 (0.2) | 30 (0.2) | 0.000 |
| **Uterine diseases** |  |  |  |  |
| Endometrial polyp |  | 2260 (12.6) | 2260 (12.6) | 0.000 |
| Endometriosis |  | 1342 (7.5) | 1342 (7.5) | 0.000 |
| Pelvic inflammatory disease |  | 1284 (7.2) | 1284 (7.2) | 0.000 |
| Uterine myoma |  | 2243 (12.5) | 2243 (12.5) | 0.000 |
| Polycystic ovary syndrome |  | 2134 (11.9) | 2134 (11.9) | 0.000 |
| **Comorbidities** |  |  |  |  |
| Headache/migraine |  | 560 (3.1) | 560 (3.1) | 0.000 |
| Hypertension |  | 382 (2.1) | 382 (2.1) | 0.000 |
| Diabetes |  | 481 (2.7) | 481 (2.7) | 0.000 |
| Renal disease |  | 89 (0.5) | 89 (0.5) | 0.000 |
| Gastrointestinal disorder |  | 9256 (51.8) | 9256 (51.8) | 0.000 |
| Thyroid disorder |  | 3378 (18.9) | 3378 (18.9) | 0.000 |
| Autoimmune disease |  | 2255 (12.6) | 2255 (12.6) | 0.000 |
| **Comedications** |  |  |  |  |
| Antidepressants |  | 358 (2.0) | 358 (2.0) | 0.000 |
| Antipsychotics |  | 80 (0.4) | 80 (0.4) | 0.000 |
| Z-drugs |  | 219 (1.2) | 219 (1.2) | 0.000 |
| Anticonvulsants |  | 377 (2.1) | 377 (2.1) | 0.000 |
| Folic acid |  | 866 (4.8) | 866 (4.8) | 0.000 |
| **Healthcare utilization** |  |  |  |  |
| Number of outpatient visits, mean (SD) |  | 12.6 (6.3) | 12.6 (6.3) | 0.000 |
| Number of hospitalizations, mean (SD) |  | 0.1 (0.2) | 0.1 (0.2) | 0.000 |
| Number of emergency room visits, mean (SD) |  | 0.1 (0.3) | 0.1 (0.2) | 0.000 |

**Abbreviations:** ET, embryo transfer; aSMD, absolute standardized mean difference; FET, frozen embryo transfer;

^a^ Others included cases where two or more cycle types (IVF, ICSI, or FET) were combined within the same cycle.

# **Supplementary Table S6. Sensitivity analyses**

|  | **Users** | | **Non-users** | | **Relative risk (95% CI)** | |
| --- | --- | --- | --- | --- | --- | --- |
|  | **No. of events/total** | **Risk (%)** | **No. of events/total** | **Risk (%)** | **Crude** | **PS-adjusted** |
| **Clinical pregnancy** | | | | | | |
| **Main analysis** | 10 324/28 649 | 36.0 | 21 917/61 433 | 35.7 | 1.01 (0.99, 1.03) | 1.02 (1.00, 1.05) |
| **Sensitivity analyses** |  |  |  |  |  |  |
| ATT-PS matching | 9642/26 369 | 36.6 | 9360/26 369 | 35.5 | 1.01 (0.99, 1.03) | 1.03 (1.01, 1.05) |
| Redefinition of LMP | 10 347/28 716 | 36.0 | 21 920/61 433 | 35.7 | 1.01 (0.99, 1.03) | 1.02 (1.01, 1.04) |
| No washout period | 12 797/36 876 | 34.7 | 30 956/89 940 | 34.4 | 1.01 (0.99, 1.03) | 1.01 (0.99, 1.03) |
| Concomitant use of other medications for conscious sedation during the IVF cycle^a^ | 9350/25 479 | 36.7 | 18 579/50 892 | 36.5 | 1.01 (0.99, 1.03) | 1.02 (1.00, 1.05) |
| **Miscarriage** | | | | | | |
| **Main analysis** | 1535/10 324 | 14.9 | 3329/21 917 | 15.2 | 0.98 (0.93, 1.04) | 0.99 (0.91, 1.08) |
| **Sensitivity analyses** |  |  |  |  |  |  |
| ATT-PS matching | 1416/9642 | 14.7 | 1385/9360 | 14.8 | 0.98 (0.93, 1.04) | 0.99 (0.93, 1.06) |
| Redefinition of LMP | 1543/10 347 | 14.9 | 3329/21 920 | 15.2 | 0.98 (0.93, 1.04) | 0.99 (0.94, 1.05) |
| No washout period | 1934/12 797 | 15.1 | 4789/30 956 | 15.5 | 0.98 (0.93, 1.03) | 1.00 (0.95, 1.05) |
| Concomitant use of other medications for conscious sedation during the IVF cycle^a^ | 1408/9350 | 15.1 | 2800/18 579 | 15.1 | 1.00 (0.94, 1.06) | 1.00 (0.94, 1.07) |
| **Live birth** | | | | | | |
| **Main analysis** | 8575/8634 | 99.3 | 18 107/18 242 | 99.3 | 1.00 (1.00, 1.00) | 1.00 (1.00, 1.00) |
| **Sensitivity analyses** |  |  |  |  |  |  |
| ATT-PS matching | 8030/8085 | 99.3 | 7782/7835 | 99.3 | 1.00 (1.00, 1.00) | 1.00 (1.00, 1.00) |
| Redefinition of LMP | 8588/8647 | 99.3 | 18 107/18 242 | 99.3 | 1.00 (1.00, 1.00) | 1.00 (1.00, 1.00) |
| No washout period | 10 581/10 661 | 99.3 | 25 486/25 674 | 99.3 | 1.00 (1.00, 1.00) | 1.00 (1.00, 1.00) |
| Concomitant use of other medications for conscious sedation during the IVF cycle^a^ | 7756/7811 | 99.3 | 15 388/15 508 | 99.2 | 1.00 (1.00, 1.00) | 1.00 (1.00, 1.00) |
| **Preterm birth** | | | | | | |
| **Main analysis** | 1150/8634 | 13.2 | 2122/18 242 | 11.6 | 1.15 (1.07, 1.22) | 1.10 (0.99, 1.21) |
| **Sensitivity analyses** |  |  |  |  |  |  |
| ATT-PS matching | 1060/8085 | 13.1 | 942/7835 | 12.0 | 1.15 (1.07, 1.22) | 1.09 (1.00, 1.18) |
| Redefinition of LMP | 1151/8647 | 13.3 | 2122/18 242 | 11.6 | 1.14 (1.07, 1.22) | 1.10 (1.02, 1.17) |
| No washout period | 1423/10 661 | 13.4 | 2976/25 674 | 11.6 | 1.15 (1.09, 1.22) | 1.11 (1.04, 1.18) |
| Concomitant use of other medications for conscious sedation during the IVF cycle^a^ | 1030/7811 | 13.2 | 1845/15 508 | 11.9 | 1.11 (1.03, 1.19) | 1.08 (1.00, 1.16) |

**Abbreviations:** PS, propensity score; ATT, average treatment effect on the treated; LMP, last menstrual period

^a^ propofol or opioids^1^

^1^ Ulyatt JM, Martins da Silva SJ. Conscious Sedation and Analgesia for Oocyte Retrieval. In: Cutting R, Metwally M, eds. Manual of Oocyte Retrieval and Preparation in Human Assisted Reproduction. Cambridge University Press; 2022:24-36.

# **Supplementary Table S7. Supplementary analysis of ectopic pregnancy rate**

|  | **Users** | | **Non-users** | | **Relative risk (95% CI)** | |
| --- | --- | --- | --- | --- | --- | --- |
|  | **No. of Events/total** | **Risk (%)** | **No. of Events/total** | **Risk (%)** | **Crude** | **PS-adjusted** |
| Ectopic pregnancy | 345/28 649 | 1.2 | 758/61 433 | 1.2 | 0.98 (0.86, 1.11) | 0.94 (0.83, 1.08) |

**Abbreviations:** PS, propensity score

# **Supplementary File S1. Algorithms for identifying pregnancy episodes after IVF cycles and definition of IVF outcomes**

- **The last menstrual period (LMP)**: For pregnancies conceived via IVF cycles, the LMP is generally estimated as 2 weeks (14 days) before the oocyte retrieval date. Given that embryo transfer occurs after 3–5 days of culture following oocyte retrieval, in the main analysis, LMP was estimated by subtracting 17 days from the embryo transfer date, and in the sensitivity analysis, by subtracting 19 days from the embryo transfer date.
- **Hierarchy of pregnancy episode:
  Stillbirth > Live birth > Induced abortion > Ectopic pregnancy > Miscarriage**

  To identify pregnancy outcomes, pregnancy episodes were defined based on relevant time windows and procedure or diagnosis codes. If multiple episodes overlapped, they were merged into a single episode according to the hierarchy described above. The episode end date was defined as the date of the last relevant code recorded within the corresponding time window. We refined this algorithm by adapting a previously developed method applicable to the Korean claims database.^1,2^ These operational definitions were also validated through review by obstetrics and gynecology specialists.
- **Stillbirth:** ≥ 1 visit with stillbirth diagnosis code OR related procedure code between 141 days and 308 days after LMP
- **Live birth:** ≥ 1 visit with delivery procedure code between 141 days and 308 days after LMP
- **Miscarriage:** ≥ 1 visit with spontaneous abortion diagnosis code OR procedure code, between embryo transfer date and 140 days after LMP
- **Induced abortion:** ≥ 1 visit with induced abortion diagnosis code OR ≥ 1 visit with related procedure code between embryo transfer and 168 days after LMP
- **Ectopic pregnancy:** ≥ 1 visit with ectopic pregnancy diagnosis code OR procedure code between embryo transfer and 91 days after LMP

  After identifying pregnancy episodes using the algorithm, we defined clinical pregnancy and preterm birth as follows.
- **Clinical pregnancy:** stillbirth OR live birth OR induced abortion OR cases with specific ultrasound procedure code for gestational sac confirmation (EB512) or fetal heartbeat (EB511) within 4 weeks after embryo transfer.

  However, the following cases were excluded:

1) No pregnancy episode
2) Ectopic pregnancy
3) Miscarriage in which the pregnancy end date was the same or earlier than the ultrasound procedure date
4) Presence of assisted reproductive technology related procedure codes (IVF, ICSI, IUI, oocyte retrieval, embryo transfer, or thawing) between the ultrasound procedure date and the pregnancy episode end date.

- **Preterm birth:** Live births in which the pregnancy end date occurred between 141 days and 252 days after LMP

^1^ Jung Y-S, Song Y-J, Keum J, Lee JW, Jang EJ, Cho S-K, Sung Y-K, Jung S-Y. Identifying pregnancy episodes and estimating the last menstrual period using an administrative database in Korea: an application to patients with systemic lupus erythematosus. Epidemiol Health 2023;46: e2024012.

^2^ Cho Y, Choi EY, Lee H, Noh Y, Han JY, Choe SA, Kim H, Shin JY. Development and Validation of Gestational Age Estimation Algorithms for Nonlive Births in Administrative Healthcare Databases. Epidemiology 2026;37: 336-344.

# **Supplementary File S2. Utilization trend of benzodiazepine for infertile women undergoing IVF cycle**

(1) **Distribution of prescription stratified by administration type**

|  | **Prescription timing relative to embryo transfer date (day)** | | | | | | | | | | | | | | | | | |
| --- | --- | --- | --- | --- | --- | --- | --- | --- | --- | --- | --- | --- | --- | --- | --- | --- | --- | --- |
|  | **-17** | **-16** | **-15** | **-14** | **-13** | **-12** | **-11** | **-10** | **-9** | **-8** | **-7** | **-6** | **-5** | **-4** | **-3** | **-2** | **-1** | **0** |
| Inj | 14 | 21 | 17 | 20 | 7 | 15 | 9 | 11 | 17 | 41 | 153 | 119 | 4291 | 5657 | 15 966 | 996 | 6 | 111 |
| (%) | 0.05 | 0.08 | 0.06 | 0.07 | 0.03 | 0.05 | 0.03 | 0.04 | 0.06 | 0.15 | 0.56 | 0.43 | 15.62 | 20.59 | 58.12 | 3.63 | 0.02 | 0.40 |
| Oral | 79 | 59 | 56 | 57 | 28 | 40 | 54 | 56 | 31 | 36 | 125 | 107 | 119 | 167 | 296 | 52 | 38 | 4235 |
| (%) | 1.40 | 1.05 | 0.99 | 1.01 | 0.50 | 0.71 | 0.96 | 0.99 | 0.55 | 0.64 | 2.22 | 1.90 | 2.11 | 2.96 | 5.25 | 0.92 | 0.67 | 75.16 |
| Total | 93 | 80 | 73 | 77 | 35 | 55 | 63 | 67 | 48 | 77 | 278 | 226 | 4410 | 5824 | 16 262 | 1048 | 44 | 4346 |
| (%) | 0.28 | 0.24 | 0.22 | 0.23 | 0.11 | 0.17 | 0.19 | 0.20 | 0.14 | 0.23 | 0.84 | 0.68 | 13.32 | 17.59 | 49.12 | 3.17 | 0.13 | 13.13 |

(2) **Main indication for benzodiazepine use stratified by administration type**

| **(2-1) main indication for injection (total: N=35 115)** | | | |
| --- | --- | --- | --- |
| **No.** | **ICD-10 code (three-digit)** | **Details** | **Frequencies (%)** |
| 1 | N97 | Female infertility | 22 144 (63.1) |
| 2 | Z31 | Procreative management | 12 021 (34.2) |
| 3 | E28 | Ovarian dysfunction | 162 (0.5) |
| 4 | N80 | Endometriosis | 130 (0.4) |
| 5 | N84 | Polyp of female genital tract | 123 (0.4) |
| 6 | D25 | Leiomyoma of uterus | 85 (0.2) |
| 7 | N85 | Other noninflammatory disorders of uterus, except cervix | 49 (0.1) |
| 8 | N96 | Recurrent miscarriage | 35 (0.1) |
| 9 | N70 | Salpingitis and oophoritis | 34 (0.1) |
| 10 | K21 | Oesophagitis | 28 (0.1) |

| **(2-2) main indication for oral (total: N=8077)** | | | |
| --- | --- | --- | --- |
| **No.** | **ICD-10 code (three-digit)** | **Details** | **Frequencies (%)** |
| 1 | N97 | Female infertility | 4150 (51.4) |
| 2 | Z31 | Procreative management | 2831 (35.1) |
| 3 | F32 | Depressive episode | 209 (2.6) |
| 4 | F41 | Other anxiety disorders | 209 (2.6) |
| 5 | N84 | Polyp of female genital tract | 93 (1.2) |
| 6 | F43 | Reaction to sever stress, and adjustment disorders | 57 (0.7) |
| 7 | F33 | Recurrent depressive disorder | 46 (0.6) |
| 8 | F31 | Bipolar affective disorder | 35 (0.4) |
| 9 | G47 | Sleep disorders | 33 (0.4) |
| 10 | H81 | Disorders of vestibular function | 23 (0.3) |

**Abbreviations:** LMP, last menstrual period; ICD-10, International Classification of Diseases 10^th^ Revision
